# Adult-Onset Still’s Disease with Distinctive Skin Signs and Elevated LDH

**DOI:** 10.31138/mjr.200224.aod

**Published:** 2024-05-21

**Authors:** Eleni Klimi, Evaggelia Kataxaki

**Affiliations:** 1 Department of Dermatology, Thriassio General Hospital, Magoula, Greece,; 2 Department of Rheumatology, Thriassio General Hospital Magoula, Greece

**Keywords:** adult, onset, Still’s disease, distinctive, skin

Adult-onset Still’s disease (AOSD) is a rare, inflammatory disorder characterised by the appearance of fever and salmon-patch like lesions, that may affect multiple organs and joints, including the liver and spleen. The disease responds to the administration of steroids, although biological agents have been used in the treatment of recalcitrant cases. Recently, some cases presenting atypical skin signs have been reported in literature. Herein, we describe such a case which, apart from the typical salmon-patch rash, presents erythematous urticarial papules lesions that coalesce to form plaques in the extremities. In addition, elevated LDH in the serum is a laboratory finding suggestive of a worse prognosis.

**Figure 1.**
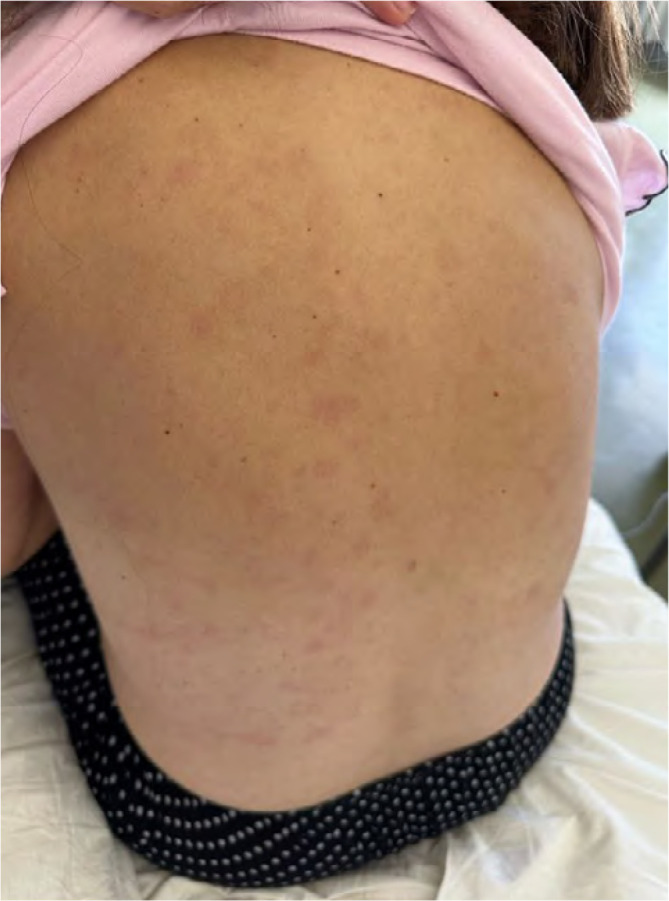
Salmon-like patch lesions on the back.

**Figure 2.**
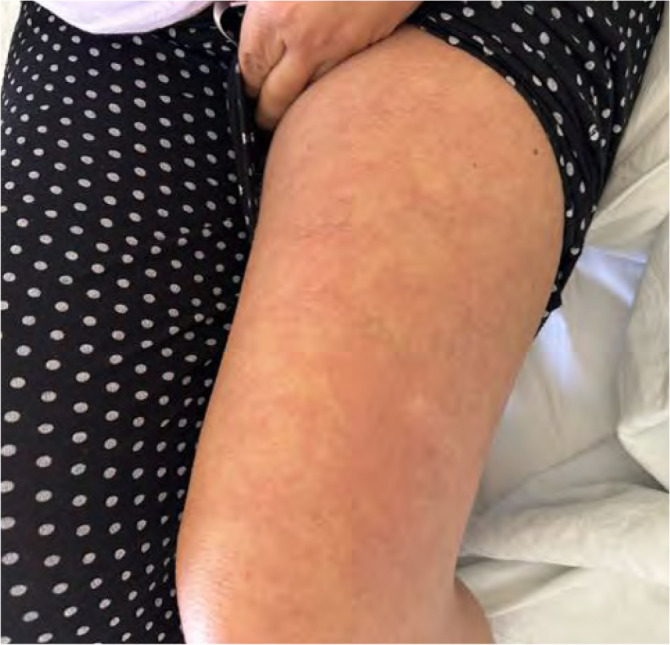
Erythematous urticarial papules that coalesce to form plaques on the left leg.

A 45-year-old woman was hospitalised in the Department of Internal Medicine with a 39^o^C fever and a rash that had appeared 4 days prior to the consultation. No intake of medications was reported by the patient. Their family history includes systemic lupus erythematosus in the mother and coeliac disease in two of the children – a son and a daughter. A dermatological consultation was requested. The clinical examination revealed salmon-like patches on the back (**Figure 1**) and erythematous papules that coalesce to form urticarial-like plaques on both thighs (**Figure 2**). Pruritus and arthralgias were not reported. The laboratory findings were leukopenia, abnormal hepatic enzymes, abnormal ESR, abnormal CRP. Haemocultures and urine cultures were sterile. The strep test from the throat and antistreptolysin-O (ASTO) were negative.

Serology for Ebstein-Barr, Coxsackie, Echo, HIV1,2 and COVID-19 also proved negative. Anti-nuclear antibody and rheumatoid factor were negative (**Table 1**). Thorax radiography was normal. Echography of the abdomen revealed splenomegaly.

**Table 1.** Haematological, bitochemical, and immunological findings.

WBC3500/mm^3^ (N4000-1000 mm^3^)
ESR: 31mm/h (N<20mm/h)
C-reactive protein: 88mgr/L (N<5mgr/L)
Ferritin: 88ng/ml (N 24–336 mg/ml)
SGOT: 153U/L, (N <50 U/L)
SGPT: 192U/L, (N <50 U/L)
γGT: 187U/L, (N 8–61 IU/L)
ALP: 236U/L, (N 40–129 IU/L)
LDH: 523U/L. (N 135–225 IU/L)
Haemocultures and urine cultures sterile
The strep test from the throat and ASTO negative
Serology for Ebstein-Barr, Coxsackie, Echo, HIV1,2, and COVID-19 negative

Anti-nuclear antibody and rheumatoid factor negative

A skin biopsy was proposed but refused by the patient. A clinical suspicion of AOSD was raised and methylprednisolone at the dose of 1 mgr/kgr was started, resulting in improvement of the skin lesions and apyrexia. Two weeks later following the administration of steroids, the skin lesions and fever were completely resolved, with no recurrence during the tapering of the steroids period. Described initially by George Still in children, the adult form of the disease defined in 1971 by Eric Bywaters is a rare inflammatory condition that manifests with rash, fever, and multiple internal organ involvement.^1^ It belongs to autoinflammatory syndromes.^2^ Its aetiology is unknown, although some cases have been triggered by COVID-19 infection^3^ and/or vaccination.4 The diagnostic criteria for adult-onset Still’s disease are those of Yamaguchi^5^ (**Table 2**).

**Table 2.** Yamaguchi et al. classification criteria for AOSD.

Sensitivity: 96.2%
Specificity: 92.1%
**Major criteria:**
1. Fever 39 °C lasting at least 1 week
2. Arthralgia or arthritis for 2 weeks
3. Typical nonpruritic salmon-pink skin rash
4. Leukocytosis 10,000/mm3 with 80% polymorphonuclear cells
**Minor criteria:**
1. Sore throat
2. Lymph node enlargement
3. Hepatomegaly or splenomegaly
4. Abnormal liver function tests
5. Negative ANA and RF tests
**Exclusion criteria:**
1. Infections (especially, sepsis and infectious mononucleosis)
2. Malignancy (mainly malignant lymphoma)
3. Other rheumatic disorders (mainly polyarteritis nodosa and rheumatoid vasculitis with extraarticular features)
For diagnosis of AOSD, patient should meet “5 or more criteria, of which at least 2 should be major”

Differential diagnoses include viral exanthems, drug eruption, and lymphoma. It may be complicated by internal organ involvement and macrophage activation syndrome.^6^

In the case reported, the diagnosis of AOSD was established due to the presence of a rash, a 39^o^ C fever, splenomegaly, liver dysfunction, and the negativity of rheumatoid factor and antinuclear antibody. Thus, five criteria were present, two of them being major. Liver dysfunction exhibited by elevated enzyme levels could be attributed to hypercytokinaemia (increased levels of interleukins circulating in the serum). In addition, hyper-cytokinaemia is detected in haemophagocytic syndrome, that may complicate AOSD. No re-evaluation of the liver tests was attempted.

Recently, several cases with atypical cutaneous manifestations have been reported in literature. One such case has been reported by Jeong Woo Seong et al. It was a case of AOSD in a 77-year-old woman with erythematous coalescent figurate, oedematous popular plaques over the lower lumbar region.^7^ Bhatia R et al. reported a case of flagellate erythema in a primigravida with AOSD.^8^ Another case of AOSD with atypical skin signs is reported by Camporro Angel Fernandez et al. who reported two cases of Still’s disease, one in a paediatric patient and one in an adult with dermatomyositis-like eruption.^9^ The case reported here, in addition to the typical salmon patch–like rash on the back, presents erythematous papules which coalesce to form erythematous urticarial-like plaques on both thighs. The emergence of urticarial-like lesions might be due to the circulation of interleukins in the serum that attract polynuclear and other inflammatory cells in the epidermis. Lactate dehydrogenase (LDH) is usually elevated in patients with AOSD, yet the presence of high LDH in the serum is suggestive of a worse prognosis.^10^ Although steroids are effective treatment in most patients with AOSD, methotrexate and/or biological agents may be necessary in some cases. This case of AOSD has been reported to increase awareness about cases of AOSD which combine typical with atypical clinical signs- an urticarial-like eruption, a clinical rarity in association with high LDH, a factor suggestive of a worse prognosis.
